# Association of serum lipids and coronary artery disease with polymorphisms in the apolipoprotein AI-CIII-AIV gene cluster

**DOI:** 10.1080/2331205X.2016.1266789

**Published:** 2016-12-17

**Authors:** Himanshu Rai, Nakul Sinha, James Finn, Suraksha Agrawal, Sarabjit Mastana

**Affiliations:** ^a^ Department of Cardiology, Sanjay Gandhi Post Graduate Institute of Medical Sciences, Lucknow, UP, India; ^b^ Department of Cardiology, Sahara India Medical Institute, Lucknow, UP, India; ^c^ Human Genomics Laboratory, School of Sport Exercise and Health Sciences, Loughborough University, LoughboroughLE11 3TU, UK; ^d^ Department of Medical Genetics, Sanjay Gandhi Post Graduate Institute of Medical Sciences, Lucknow, UP, India; ^e^ Roosevelt University, USA

**Keywords:** apolipoprotein (Apo) AI-CIII-AIV gene cluster, single nucleotide polymorphisms (SNPs), serum lipids, coronary artery disease (CAD), linkage disequilibrium, haplotypes

## Abstract

Genetic variants are considered as one of the main determinants of the concentration of serum lipids and coronary artery disease (CAD). Polymorphisms in the Apolipoprotein (Apo) AI-CIII-AIV gene cluster has been known to affect the concentrations of various lipid sub-fractions and the risk of CAD. The present study assessed associations between polymorphisms of the Apo AI-CIII-AIV gene cluster, [ApoA-I,-75G > A, (rs1799837); ApoC-III 3238C > G, (*SstI*), (rs5128) and ApoA-IV, Thr347Ser(347A > T), (rs675)] with serum lipids and their contributions to CAD in North Indian population. We recruited age, sex matched, 200 CAD patients and 200 healthy controls and tested them for fasting levels of serum lipids. We genotyped selected polymorphisms using polymerase chain reaction-restriction fragment length polymorphism. There were no statistically significant association of selected polymorphisms (or their combinations) with CAD even after employing additive, dominant and recessive models. However there was significant association of selected polymorphisms with various lipid traits amongst the control cohort (*p* < 0.05). Mean levels of high density lipoprotein cholesterol and triglycerides were found to be significantly higher among controls carrying at least one mutant allele at ApoA1-75G > A (*p* = 0.019) and ApoCIII *SstI* (*p* < 0.001) polymorphism respectively. Our study observed that the selected polymorphisms in the ApoAI-CIII-AIV gene cluster although significantly affect various lipid traits but this affect does not seem to translate into association with CAD, at least among North Indian population.

## 1. Introduction

Coronary artery disease (CAD) accounts for roughly one half of all cardiovascular deaths and is a major cause of morbidity and mortality around the world. Both genetic and environmental factors play a crucial role in the causation of CAD. Current evidences suggest that positive family history of CAD and different alterations in lipid metabolism, including high low density lipoprotein cholesterol (LDL-c) and low high density lipoprotein cholesterol (HDL-c) levels (both separately and jointly), high triglyceride (TG) levels, high apoB levels, high lipoprotein (a) (Lp(a)) levels, are important risk factors for CAD. The lipid abnormalities significantly contribute to the risk of developing premature CAD. These lipid abnormalities also have genetic determinants (Sankaranarayanan, Chakraborty, & Boerwinkle, 1999). One of the commonly studied lipid gene cluster is the Apolipoprotein (Apo) AI-CIII-AIV gene cluster (Agrawal & Mastana, 2014). This group of genes is located on chromosome 11q23–q24. Several polymorphic forms of the genes in this cluster not only can alter plasma levels of various lipids but can affect expression of other genes and/or modulate the action of different proteins within the human body, which can lead to higher chance of developing premature CAD.

ApoA-I, which is mainly synthesized in the liver and the small intestine, is an obligatory activator of lecithin cholesterol acyltransferase (LCAT). It is a major apolipoprotein component of HDL-c, and is known to promote cholesterol efflux from macrophages and return it to the liver for excretion, a process commonly known as “reverse cholesterol transport” (Lewis & Rader, 2005). HDL-c can also prevent lipoprotein oxidation, exert anti-inflammatory actions *in vitro*, and promote cell proliferation and survival which contributes to its anti-atherogenic effects (Barter et al., 2004). This inverse relationship between plasma HDL-c levels and coronary atherosclerotic events has been demonstrated consistently by several epidemiologic studies which validates its anti-atherogenic properties. Genetic regulation of the concentrations of plasma levels of HDL-c and apoA-I are well established as a result of several twin and family studies, which have reported the heritability of HDL-c concentration to be around 40% (Pulkkinen, Viitanen, Kareinen, Lehto, & Laakso, 2000; Ordovas et al., 2002). Inter-individual variations in plasma ApoA1 and HDL are proven to be largely influenced by a common polymorphism of a guanine (G) to adenine (A) substitution (G/A) at −75 bp (rs1799837), in the promoter region of the ApoA1 gene (Jeenah, Kessling, Miller, & Humphries, 1990; Wang, Badenhop, Humphrey, & Wilcken, 1996). Non-association of HDL-c with Apo A1-75G > A polymorphism has also been reported in some studies, these contradictory results could be a result of an intricate interplay of various environmental and ethnic factors (Ordovas et al., 2002).

The liver and intestine also jointly synthesize the ApoC-III protein. Although the precise function of ApoC-III is not completely understood, there is increasing evidence that this apolipoprotein is associated with the catabolism of triglyceride-rich lipoproteins (TGRL). Animal studies have shown that ApoC-III acts as an inhibitor of the lipoprotein lipase-mediated hydrolysis of TGRL (Jong, Hofker, & Havekes, 1999), thus possibly regulating plasma triglyceride (TG) levels. A Cytosine (C) to Guanine (G) substitution at 3,238 bp (rs5128) in the ApoC-III gene (also known as *SstI* polymorphism) has been extensively studied in relation to various lipid traits. Several studies have reported an association between S2 allele of *SstI* polymorphism and elevated TG levels (Paul-Hayase et al.,^1992^; Rees et al., 1985) while others reported no association with TG levels (Price, Morris, Burgon, Donald, & Kitchin,^1986^; Marcil et al., 1996).

The precise physiological role of ApoA-IV is also not completely understood, but numerous *in vitro* studies suggest that ApoA-IV participates in several steps of “reverse cholesterol transport” by binding to peripheral cells, promoting cholesterol efﬂux and enhancing the formation of small HDL particles by activating LCAT (Steinmetz & Utermann, 1985). In addition, it is suggested that ApoA-IV and may negatively influence lipid absorption and chylomicron assembly thus modulating body weight gain (Weinberg, 2002). Published literature suggests that Apo A-IV may also have antioxidant properties. Taken together, these evidences suggests that ApoA-IV may be considered as an anti-atherogenic factor as lower plasma levels of ApoA-IV has already been proven to be risk factor for atherogenic events (Kronenberg et al., 2000). A common Adenine (A) to Thymine (T) substitution at 347 bp in the ApoA-IV gene (also known as 347A > T, Thr347Ser polymorphism) has been shown to be associated with lower plasma levels of Apo AIV and higher incidences of CAD in some studies (Wong et al., 2003).

Several other common polymorphisms have been identified in the APOAI-CIII-AIV gene cluster, e.g. XmnI (C−2500T), and MspI (C + 83T) in Apo-AI gene (Kessling, Horsthemke, & Humphries, 1985; Paulweber, Friedl, Krempler, Humphrie, & Sandhofer, 1988; Wang et al., 1996). T−455C; in Apo CIII gene (Dammerman, Sandkuijl, Halaas, Chung, & Breslow,^1993^; Groenendijk et al., 1999), PstI polymorphism in the Apo AI-CIII intragenic region (Vavatsi et al., 1995) and Gln360His in Apo AIV gene (Kretowski et al., 2006) have been studied for their effect on various lipid traits and contribution to CAD. APOAI-CIII-AIV gene cluster polymorphisms that we aimed to study, i.e. Apo AI-75G > A polymorphism, Apo CIII- *SstI* polymorphism and Apo AIV-Thr347Ser polymorphism are among the important ones and studies of these loci among Indians populations are limited (Singh, Singh, Gaur, & Kaur, 2007; Singh, Singh, Kaur, & Grewal, 2008). Moreover, except for few sporadic studies, the distributions of apolipoprotein polymorphisms have mainly been studied in European populations, the results of which may or may not translate among Indians. Also, it is well known that differences among populations in the relative frequency of susceptibility genotypes or environmental exposure will contribute to differences in the utility of a genotype for predicting a trait within a particular population.

Our primary objective therefore was to test the association between polymorphisms of the APOAI-CIII-AIV gene cluster, namely: (i) Apo AI-75G > A polymorphism; (rs1799837), (ii) the ApoC-III 3238C > G polymorphism (*SstI* polymorphism); (rs5128) and (iii) 347A > T polymorphism (also known as Thr347Ser polymorphism); (rs675) in ApoA-IV gene on plasma levels of various lipid traits and their role in the pathogenesis of CAD among North Indian population from Uttar Pradesh (UP).

## 2. Materials and methods

### 2.1. Subjects

This study was carried out by departments of Cardiology and Medical Genetics at Sanjay Gandhi Post Graduate Institute of Medical Sciences (SGPGIMS), Lucknow, India in collaboration with School of Sport Exercise and Health Sciences, Loughborough University, Leicestershire, UK. The study was approved by local ethics committees of SGPGIMS, Lucknow and Loughborough University.

We prospectively included 200 proven CAD patients and 200 healthy case controls in the present study after obtaining the written informed consent. Proven CAD was defined as the detection of at least 50% or more stenosis in one or more native coronary arteries of the patient, verified through coronary angiography. Patients who experienced even a single symptom of rest angina during last 6 weeks were excluded. Demographics, anthropometrics and clinical history of all subjects were collected using a uniform clinical proforma. Diabetes, hypertension, smoking and family history of premature CAD was determined in the subjects as per standard definitions. The healthy controls included in the present study were sex matched and had no known history of ischemic heart disease, endocrine or metabolic disorders. They were selected after administration of an exercise cardiac stress test (treadmill test) or a physiologic stress test (Dobutamine Stress echocardiogram) to negate the possibility of an underlying CAD. All selected cases and controls were of North Indian ethnicity and were residents of Uttar Pradesh, since last five generations.

Three millilitres of EDTA whole blood was collected for DNA extraction. DNA was extracted from whole blood by using commercially available Qiagen kits (QIAamp DNA Mini Kit; QiagenInc. Valencia, CA USA) as per the manufacturers protocol.

### 2.2. Lipid estimations

Three millilitres of fasting blood from each subject was drawn for lipid profile. Direct estimation of Total cholesterol (TC), and HDL-c levels was done from fasting serum samples employing CHOD-PAP method. Triglycerides (TG) were also directly estimated from fasting serum samples employing a GPO-PAP method. All lipid estimations were done using RX Imola benchtop clinical chemistry Analyzer (Randox Laboratories Ltd). Low and very low density lipoprotein cholesterols (LDL-c and VLDL-c) were calculated employing the Friedewald’s formula (Friedewald, Levy, & Fredrickson, 1972), i.e. LDL-c = TC–HDL-c–(triglyceride/5) and VLDL-c = 0.20 (TG), respectively.

### 2.3. Genotyping techniques

The samples were genotyped for these single nucleotide polymorphisms (SNP’s) using polymerase chain reaction-restriction fragment length polymorphism (PCR-RFLP) method. Genotyping conditions were used as described in previously published literature (Hixson & Powers, 1991; Paul-Hayase et al., 1992). Genotypes were determined on the basis of presence or absence of bands on the gel photographs. Approximately 10% of samples were repeated randomly to assess the reliability of PCR-RFLP methods and genotyping. All genotyping was done without the knowledge of disease status. The genotypes were scored independently by two researchers and the allele frequencies were calculated by allele counting method.

### 2.4. Statistical analysis

Statistical analysis was carried out using the computer packages EXCEL and SPSS^®^ for WINDOWS softwares (version 16.0; SPSS^®^ Inc, Chicago IL). Independent t-tests were used to analyse differences between the means of continuous variables. Discrete variables, genotype distribution and Hardy–Weinberg equilibrium (HWE) were tested using *χ* ^2^ test with Yates’s correction. Odds Ratios (OR’s) 95% confidence intervals (CI) and associated chi-squares were calculated for genotypes and alleles. Haplotype analysis was performed using Arlequin^®^ software (version 3.11) (Excoffier, Laval, & Schneider, 2005). Two-tailed p values of <0.05 were considered to be statistically significant.

## 3. Results

The patient and control groups were age, sex and ethnicity matched (*p* > 0.05). No significant difference was observed for dietary habits, though patient group reported a higher number of non-vegetarians (46 vs. 39%, *p* = 0.19, NS).

Statistically significantly differences were observed between the two groups for some conventional risk factors for CAD (Table 1). There were more smokers among patients (34.5%) as opposed to in controls (22.0%) (*p* = 0.007); more hypertensives among patients (28.5%) vs. in controls (16.0%) (*p* = 0.004); higher number of diabetics among patients (24.0%) vs. controls (6.5%) (*p* < 0.001) and 16.0% of the patients had a family history of premature CAD compared to controls (6.5%) (*p* = 0.004) (Table 1).

**Table 1.** Baseline characteristics in patient/control groups

		CAD patients (*n* = 200)	Controls (*n* = 200)	Odds ratio (OR)/*t*	*p*-value
Age (years)	Mean ± SD	49.65 ± 11.59	48.53 ± 13.38	0.89!	0.37
Males	*n* (%)	169 (84.5%)	163 (81.5%)	1.12	0.50
Smokers	*n* (%)	69 (34.5%)	44 (22.0%)	1.34	0.007^*^
Non vegetarians	*n* (%)	92 (46.0%)	78 (39.0%)	1.15	0.19
Family history of premature CAD	*n* (%)	32 (16.0%)	13 (6.5%)	1.50	0.004^*^
Hypertension	*n* (%)	57 (28.5%)	32 (16.0%)	1.39	0.004^*^
Diabetes mellitus	*n* (%)	48 (24.0%)	13 (6.5%)	1.75	<0.001^*^
Alcohol consumers	*n* (%)	68 (34.0%)	51 (25.5%)	1.22	0.08
Total cholesterol (mg/dl)	Mean ± SD	173.84 ± 43.49	135.22 ± 31.24	10.97!	<0.001^*^
Triglycerides (mg/dl)	Mean ± SD	193.99 ± 102.41	140.65 ± 62.19	6.29!	<0.001^*^
HDL-c (mg/dl)	Mean ± SD	32.72 ± 13.17	27.73 ± 9.89	4.28!	<0.001^*^
LDL-c (mg/dl)	Mean ± SD	105.53 ± 39.92	80.75 ± 24.23	7.50!	<0.001^*^
VLDL-c (mg/dl)	Mean ± SD	35.45 ± 14.30	28.13 ± 12.44	5.46!	<0.001^*^

Abbreviations used: HDL-c—high density lipoprotein cholesterol; LDL-c and VLDL-c—low and very low density lipoprotein cholesterol.

^*^ *p*-value of <0.05 was considered to be statistically significant; !—*t* test statistics.

Eighty-one percent of patients (*n* = 162) were on lipid lowering therapy (at the time of their inclusion), the patient group still had markedly higher mean levels of various serum lipid parameters compared to the control group (*p* < 0.001). Similarly mean levels of TC/HDL and LDL/HDL ratios were also significantly higher in patients when compared to controls (*p* = 0.002) (Data not shown).

Genotype frequencies in patients and controls for all of the studied SNP’s did not differ from that expected for Hardy–Weinberg proportions. We found no significant differences in genotype and allele frequencies between patient and control groups for ApoAI-75G > A, ApoC-III 3238C > G (*SstI*) and ApoA-IV 347A > T (Thr347Ser) polymorphisms (*p* > 0.05), (Table 2). Even after employing additive, dominant and recessive models, no statistically significant association of these polymorphisms with CAD was observed in this population (Table 2) though many odds ratios containing risk alleles were above 1.

**Table 2.** Apo AI-CIII-AIV gene cluster polymorphisms in CAD patients vs. controls

	CAD patients (*n* = 200)	Controls (*n* = 200)	OR (95% CI)	*χ*^2^	*p*-value
*Apo AI, -75G → A polymorphism*					
AA (mutant)	12 (6.0%)	7 (3.5%)	1.28 (0.89–1.83)	0.88	0.35
GA (Heterozygous)	71 (35.5%)	75 (37.5%)	0.96 (0.78–1.18)	0.09	0.75
GG (Wild type)	117 (58.5%)	118 (59.0%)	0.99 (0.81–1.20)	0.01	0.92
AA vs. GG	12/117	07/118	1.27 (0.88–1.83)	0.78	0.38
GA vs. GG	71/117	75/118	0.98 (0.79–1.20)	0.01	0.90
AA + GA vs. GG (dominant model)	83/117	82/118	1.01 (0.83–1.23)	0.01	0.92
AA vs. GA + GG (recessive model)	12/188	07/193	1.28 (0.89–1.83)	0.88	0.35
Allele A (frequency)	0.24	0.22	1.06 (0.77–1.45)	0.02	0.87
Allele G (frequency)	0.76	0.78			
*Apo CIII, 3238C → G polymorphism (SstI Polymorphism)*					
GG (mutant)	21 (10.5%)	20 (10.0%)	1.03 (0.75–1.40)	0.02	0.87
CG (Heterozygous)	74 (37%)	68 (34.0%)	1.07 (0.87–1.30)	0.27	0.60
CC (Wild type)	105 (52.5%)	112 (56.0%)	0.93 (0.77–1.13)	0.36	0.55
GG vs. CC	21/105	20/112	1.05 (0.76–1.47)	0.02	0.87
CG vs. CC	74/105	68/112	1.08 (0.87–1.33)	0.34	0.56
GG + CG vs. CC (dominant model)	95/105	88/112	1.07 (0.88–1.30)	0.36	0.55
GG vs. CG + CC (recessive model)	21/179	20/180	1.03 (0.75–1.40)	0.02	0.87
Allele G (S2 allele frequency)	0.29	0.27	1.05 (0.78–1.42)	0.02	0.87
Allele C (S1 allele frequency)	0.71	0.73			
*Apo AIV, 347A → T polymorphism (Thr347Ser Polymorphism)*					
TT (mutant)	30 (15.0%)	19 (9.5%)	1.26 (0.99–1.62)	2.33	0.13
AT (Heterozygous)	81 (40.5%)	75 (37.5%)	1.06 (0.87–1.30)	0.26	0.61
AA (Wild type)	89 (44.5%)	106 (53.0%)	0.84 (0.69–1.03)	2.56	0.10
TT vs. AA	30/89	19/106	1.34 (1.02–1.76)	3.21	0.07
AT vs. AA	81/89	75/106	1.14 (0.92–1.41)	1.13	0.28
TT + AT vs. AA (dominant model)	111/89	94/106	1.19 (0.97–1.45)	2.56	0.11
TT vs. AT + AA (recessive model)	30/170	19/181	1.26 (0.99–1.62)	2.33	0.13
Allele T (347Ser allele frequency)	0.35	0.28	1.17 (0.88–1.55)	0.83	0.36
Allele A (Thr347 allele frequency)	0.65	0.72			

^*^ *p*-value of <0.05 was considered to be statistically significant.

Haplotype analysis also did not show any statistically significant association of any of the possible haplotypes with CAD (*p* > 0.05) (Table 3), though some of the haplotypes were more frequent in patients.

**Table 3.** Haplotype analysis for association with CAD

Haplotypes^**^	Patients (*n* = 200)	Controls (*n* = 200)	OR	95% CI	*p*-value
A-C-A	83 (20.75%)	83 (20.75%)	1	0.71–1.40	1
G-C-T	32 (8.00%)	24 (6.00%)	1.36	0.79–2.35	0.33
G-C-A	164 (41.00%)	183 (45.75%)	0.82	0.62–1.09	0.20
A-C-T	5 (1.25%)	2 (0.5%)	2.52	0.48–13.06	0.45
G-G-T	97 (24.25%)	83 (20.75%)	1.22	0.88–1.70	0.27
A-G-T	7 (1.75%)	4 (1.00%)	1.76	0.51–6.07	0.54
G-G-A	12 (3.00%)	21 (5.25%)	0.56	0.27–1.15	0.15

^*^ *p*-value of <0.05 was considered to be statistically significant.

^**^ The order of SNPs within each haplotype is Apo AI (-75G/A polymorphism); Apo CIII (SSTL polymorphism); Apo AIV (T347S polymorphism).

We also assessed the role of all possible genotypic combinations in CAD. Possible genotypic combinations in two polymorphisms at a time, revealed that the subjects who have at least one mutant allele for the ApoA-I or ApoA-IV genes, are at a comparatively higher risk for CAD than other genotypic combinations (*p* = 0.03, OR = 1.33) (supplementary Table 2). However, we did not find any clear cut trend for the same when we examined genotypic combinations among all the three SNP’s (*p* > 0.05) (supplementary Table 2).

We investigated the association of these SNP’s with various lipid traits in patients and controls. We chose to assess the trends only for TC, TG and HDL-c values, as they were directly estimated as opposed to other traits (e.g. LDL-c, VLDL-c values and TC/HDL and LDL/HDL ratios) which were calculated values. As expected no difference was seen among the patient sub group owing to the effect of the lipid lowering therapy which was ongoing in a large majority of patients (~81%, as discussed earlier). On the other hand, after employing additive, dominant and recessive models in the control group we found conclusive evidence of the association of the studied SNP’s with lipid traits (Table 4). Significantly higher mean HDL-c values were seen among mutants for Apo AI-75G > A polymorphism, as confirmed after comparing them using additive and dominant models (*p* = 0.042 and 0.019, respectively) (Table 4). G allele carriers for Apo CIII-*SstI* polymorphism were associated with higher TG levels, as evident by significant p values on comparisons using additive (*p* = 0.002 and 0.007), dominant (*p* < 0.001) and recessive models (*p* = 0.014) (Table 4). Heterozygotes (AT) at Apo AIV, Thr347Ser polymorphism had lower mean HDL-c values compared to wild type genotype (AA) in additive model (*p* = 0.012) (Table 4).

**Table 4.** Lipid levels among disease free controls according to different genetic models

	*n*	Serum lipid levels Mean ± SD (SEM)	95% CI (diff)	*p*-value
*Apo AI,* -*75G* > *A polymorphism*				
*AA* vs*. GG*	*7 vs. 118*			
TC (mg/dl)		152.14 ± 45.26 (17.11) vs. 132.47 ± 30.24 (2.78)	−22.22 to 61.56	0.298
TG (mg/dl)		147.71 ± 51.24 (19.37) vs. 135.59 ± 56.48 (5.20)	−35.45 to 59.69	0.565
HDL-c (mg/dl)		34.29 ± 8.48 (3.21) vs. 27.19 ± 9.58 (0.88)	−0.78 to 14.96	0.071
*GA vs. GG*	*75 vs. 118*			
TC (mg/dl)		137.96 ± 31.09 (3.59) vs. 132.47 ± 30.24 (2.78)	−3.49 to 14.46	0.229
TG (mg/dl)		147.49 ± 65.20 (7.53) vs. 135.59 ± 56.48 (5.20)	−6.19 to 29.99	0.196
HDL-c (mg/dl)		30.23 ± 27.19 (1.18) vs. 27.19 vs. 9.58 (0.88)	0.12 to 5.95	0.042^*^
*AA* + *GA vs. GG (dominant model)*	*82 vs. 118*			
TC (mg/dl)		139.07 ± 32.41 (3.58) vs. 132.47 ± 30.24 (2.78)	−2.26 to 15.65	0.142
TG (mg/dl)		147.51 ± 63.86 (7.05) vs. 135.59 ± 56.48 (5.20)	−5.38 to 29.22	0.176
HDL-c (mg/dl)		30.57 ± 10.11 (1.12) vs. 27.19 ± 9.58 (0.88)	0.57 to 6.19	0.019^*^
*AA vs. GA* + *GG (recessive model)*	*7 vs. 193*			
TC (mg/dl)		152.14 ± 45.26 (17.11) vs. 134.61 ± 30.61 (2.20)	−24.34 to 59.41	0.347
TG (mg/dl)		147.71 ± 51.24 (19.37) vs. 140.22 ± 60.14 (4.33)	−39.99 to 54.98	0.717
HDL-c (mg/dl)		34.28 ± 8.48 (3.21) vs. 28.37 ± 9.92 (0.71)	−1.95 to 13.77	0.117
*Apo CIII, 3238C* > *G polymorphism (SstI Polymorphism)*				
*GG vs.CC*	*20 vs. 112*			
TC (mg/dl)		138.00 ± 27.60 (6.17) vs. 134.79 ± 30.44 (2.88)	−10.73 to 17.16	0.641
TG (mg/dl)		169.00 ± 49.54 (11.08) vs. 127.70 ± 56.73 (5.36)	16.12 to 66.48	0.002^*^
HDL-c (mg/dl)		27.70 ± 9.04 (2.02) vs. 29.30 ± 10.22 (0.97)	−6.19 to 2.98	0.480
*CG vs. CC*	*68 vs. 112*			
TC (mg/dl)		135.12 ± 33.84 (4.10) vs. 134.79 ± 30.44 (2.88)	−9.58 to 10.24	0.947
TG (mg/dl)		153.15 ± 62.43 (7.57) vs. 127.70 ± 56.73 (5.36)	7.10 to 43.80	0.007^*^
HDL-c (mg/dl)		27.65 ± 9.70 (1.18) vs. 29.30 ± 10.22 (0.97)	−4.66 to 1.35	0.278
*GG* + *CG vs.CC (dominant model)*	*88 vs. 112*			
TC (mg/dl)		135.77 ± 32.40 (3.45) vs. 134.79 ± 30.44 (2.88)	−7.88 to 9.86	0.826
TG (mg/dl)		156.75 ± 59.85 (6.38) vs. 127.70 ± 56.73 (5.36)	12.61 to 45.50	<0.001^*^
HDL-c (mg/dl)		27.66 ± 9.50 (1.01) vs. 29.30 ± 10.22 (0.97)	−4.40 to 1.12	0.241
*GG vs. CG* + *CC (recessive model)*	*20 vs. 180*			
TC (mg/dl)		138.00 ± 27.60 (6.17) vs. 134.91 ± 31.68 (2.36)	−10.52 to 16.70	0.644
TG (mg/dl)		169.00 ± 49.54 (11.08) vs. 137.31 ± 60.06 (4.48)	7.11 to 56.26	0.014^*^
HDL-c (mg/dl)		27.70 ± 9.04 (2.02) vs. 28.68 ± 10.03 (0.75)	−5.42 to 3.47	0.654
*Apo AIV, 347A* > *T polymorphism (Thr347Ser Polymorphism)*				
*TT vs. AA*	*19 vs. 106*			
TC (mg/dl)		138.89 ± 26.58 (6.10) vs. 135.07 ± 30.44 (2.96)	−10.07 to 17.73	0.577
TG (mg/dl)		154.05 ± 57.60 (13.21) vs. 133.65 ± 58.08 (5.64)	−9.19 to 49.99	0.168
HDL-c (mg/dl)		31.58 ± 10.32 (2.37) vs. 29.77 ± 10.30 (1.00)	−3.49 to 7.10	0.489
*AT vs. AA*	*75 vs. 106*			
TC (mg/dl)		134.51 ± 33.69 (3.89) vs. 135.07 ± 30.44 (2.96)	−10.21 to 9.09	0.909
TG (mg/dl)		146.69 ± 62.08 (7.17) vs. 133.65 ± 58.08 (5.64)	−4.98 to 31.06	0.155
HDL-c (mg/dl)		26.13 ± 8.82 (1.02) vs. 29.77 ± 10.30 (1.00)	−6.46 to −0.82	0.012^*^
*TT* + *AT vs. AA (dominant model)*	*94 vs. 106*			
TC (mg/dl)		135.39 ± 32.29 (3.33) vs. 135.07 ± 30.44 (2.96)	−8.46 to 9.11	0.941
TG (mg/dl)		148.18 ± 60.97 (6.29) vs. 133.65 ± 58.08 (5.64)	−2.13 to 31.19	0.087
HDL-c (mg/dl)		27.23 ± 9.34 (0.96) vs. 29.77 ± 10.30 (1.00)	−5.28 to 0.20	0.069
*TT vs. AT* + *AA (recessive model)*	*19 vs. 181*			
TC (mg/dl)		138.89 ± 26.58 (6.10) vs. 134.83 ± 31.73 (2.36)	−9.44 to 17.56	0.540
TG (mg/dl)		154.05 ± 57.60 (13.21) vs. 139.05 ± 59.95 (4.46)	−13.90 to 43.90	0.294
HDL-c (mg/dl)		31.58 ± 10.32 (2.37) vs. 28.27 ± 9.85 (0.73)	−1.83 to 8.46	0.195

^*^ *p*-value of <0.05 was considered to be statistically significant.

## 4. Discussion

The present study was a prospective, single centre, case-control study to assess the risk for CAD and investigate the association with various lipid traits in subjects with genetic variations in the ApoA-I, ApoC-III and ApoA-IV genes among a sample population of North Indian ethnicity. This study was conducted in SGPGIMS: a premier, public sector, tertiary care, teaching hospital situated in Lucknow, Uttar Pradesh (UP). UP is the largest state in northern India with a total population of approximately 199 million. The data derived from the present study represents the population from UP, which primarily belong to the north Indian ancestry and also adds to the existing literature on the subject from this geo-ethnic region.

### 4.1. ApoAI-75G > A polymorphism

As discussed earlier, Apolipoprotein A-I is the major protein constituent of HDL-c and plays a crucial role in reverse cholesterol transport. Sufficient published evidence exists, which negatively associates plasma levels of both ApoA-I and HDL-c as independent risk factors for CAD (Ascaso et al., 2004; Heng, Low, & Saha, 2001). ApoAI-75G > A polymorphism influences the normal expression of Apo AI gene has also been shown to influence plasma levels of various lipid traits (Wang et al., 1996; Xu et al., 1994).

Some studies have shown the association of “A” allele (minor/mutant allele) carriers with significantly higher TG levels (Souverein, Jukema, Boekholdt, Zwinderman, & Tanck, 2005; Xu et al., 1994), whilst others have shown its no association with TG (Kamboh et al., 1999; Larson et al., 2002). In the present study, although we found higher mean TG levels amongst “A” allele carriers (in controls), but the difference were non-significant to substantiate above findings (Table 4). On the other hand, some other studies like the present study have reported a significant association of “A” allele with elevated levels of HDL-c (Paul-Hayase et al., 1992; Pagani et al., 1990). This dual role of “A” allele is more of a paradox, as it is well established that higher TG levels is a risk factor for CAD whereas higher levels of HDL-c is protective against the disease. Hypothetically, if we assume that if the presence of “A” allele in an individual raises the serum levels of both HDL-c and TG, owing to the antagonistic nature of both, it would not affect the fate of the outcome, i.e. CAD. This hypothesis could qualify as a plausible explanation for a similar outcome in the present study, where trends for both, increased levels of TG and HDL-c were seen among carriers of “A” allele (among controls) but no hint of any association of ApoAI-75G > A polymorphism with CAD was seen, even after employing different genetic models.

Our results are in contradiction with another study which reported a positive association between Apo A1-75G > A polymorphism and CAD among subjects from Punjab, Haryana and Chandigarh (Poduri, Khullar, Bahl, Sharma, & Talwar, 2009). The possible reasons for this contradiction are unclear and could be different geographical regions, uneven distribution of patients and controls in these studies, It must be stressed that even among populations of similar ethnicity and gene pool, environmental factors such as diet, stress and physical inactivity compound to an individual’s composite risk for CAD in inexplicable proportions. A multi-centric study among North Indians with a larger sample size is warranted to answer this question definitively. In another study from Northwest India, a different locus (APOA1, *Pst1* polymorphism) was also not found to be associated with CAD (Singh et al., 2007, 2008) suggesting limited role of this locus in CAD diagnosis in this region.

### 4.2. ApoCIII 3238C > G polymorphism

ApoC-III is predominantly synthesized in liver and intestine and is present on very low density lipoproteins (VLDLs) and chylomicron remnants; and to a lesser extent on high density lipoproteins (HDLs) (Breslow, 1995). The function of ApoCIII is not completely understood although it has been shown *in vitro*, to inhibit lipoprotein lipase (LPL) which limits the rate of TG hydrolysis, resulting in the delayed catabolism of TG-rich particles (Wang, McConathy, Kloer, & Alaupovic,^1985^). Furthermore, it also decreases ApoE-mediated remnant removal by displacement of ApoE from the VLDL particles *in vivo* (Aalto-Setälä et al., 1996). The presence of a polymorphic *SstI* site {S2(G) allele} in the 3′ un-translated region (UTR) (3238C > G) has been associated with elevated TG levels in various ethnic populations (Chhabra et al., 2002; Ordovas et al., 2002; Singh et al., 2007, 2008; Zeng et al., 1995).

In the present study, although we found clear cut association of genotypes (among controls) carrying either one or both mutant (G) alleles with higher TG levels, confirmed by employing codominant, dominant and recessive models of inheritance (*p* < 0.01), but frequencies of various genotypes/alleles among CAD patient and control groups were found to be comparable (*p* > 0.05), indicating no association with CAD (Tables 2–4). Our results are in concordance with other studies among North Indians (Chhabra et al., 2004; Singh et al., 2007, 2008) who reported no association of S2(G) allele with CAD, while being associated with significantly higher levels of TG. Contrary to the results reported from northern Indian populations (including our study), a recent study by Kumar and colleagues (AshokKumar et al., 2010) reported S2(G) allele to be significantly associated with CAD among South Indians. This contradiction may be because of ethnic differences, which makes South Indians more prone to CAD. This difference is reflected in the CAD prevalence rates also among South Indians which are much higher than that found in North Indians (Begom & Singh, 1995). Also since CAD is a multifactorial disease, this difference could be a result of interplay between various risk factors (both genetic and environmental), which very often provide a variety of results in subjects of different ethnicities. Initially triglyceride levels were considered to be an independent risk factor for CAD, but there is now increasing evidence that higher triglyceride levels could be more of a synergistic risk factor for CAD. It is well known that clustering of risk factors can effect in heightened chance of developing CAD in an individual, e.g. the end effects, i.e. composite risk of CAD is exponentially increased in the patients with “lipid triad”, i.e. Low HDL-c values and high LDL-c and TG values. Since this SNP effects only the serum concentrations of TG, which as we said, is a poor risk factor for CAD is probably not able to decisively affect the pathogenesis of CAD.

### 4.3. ApoA-IV Thr347Ser polymorphism

Apolipoprotein A-IV (Apo AIV) is a plasma glycoprotein synthesized in the enterocytes of the small intestine during fat absorption as a constituent of nascent chylomicrons. As evident by several *in vitro* studies, ApoA-IV is involved in several steps of the reverse cholesterol transport pathway, which removes cholesterol from peripheral cells and transports it back to the liver. Scientific evidence exists, which suggests the participation of ApoA-IV in the binding and uptake of HDL by hepatocytes (Dvorin, Gorder, Benson, & Gotto, 1986). Moreover, ApoA-IV modulates the activation of lipoprotein lipase (Goldberg, Scheraldi, Yacoub, Saxena, & Bisgaier, 1990) and the cholesteryl ester transfer protein (CETP)-mediated transfer of cholesteryl esters from HDL to low-density lipoprotein (LDL), as evident from several tissue culture studies (Guyard-Dangremont, Lagrost, & Gambert, 1994). Thus extrapolated effects of several roles of ApoA-IV may represent it as an anti-atherogenic factor. This hypothesis is supported by *in vivo* studies which have demonstrated an anti-atherogenic role for ApoA-IV (Duverger et al., 1996; Cohen et al., 1997). In a couple of studies, mice that overexpress human or mouse apoAIV and were subjected to fat rich diets demonstrated a significant reduction of aortic atherosclerotic lesions when compared to control mice. Several polymorphisms in the ApoA-IV gene have been shown to effect either an increase or a decrease in various lipid traits. The TT (homozygous mutant) genotype has been has been linked to increased risk of CAD along with lower serum concentrations of Apolipoprotein AIV among subjects from UK (Wong et al., 2003). In the present study we although found a trend of higher risk for CAD among carriers of T allele, with more OR in homozygous mutants (OR = 1.34, *p* = 0.07) than in the heterozygous ones (OR = 1.14, *p* = 0.28) but the results of comparisons were non-conclusive as evident by non-significant p values, even after employing different genetic models (Table 2).

Only some studies in the past have definitively associated this SNP with cholesterol values (Fisher, Burke, Nicaud, Ehnholm, & Humphries, 1999; Saha, Wang, Vasisht, & Kamboh, 1997) while other studies have failed to do so (Wang et al., 2003). The only published study among North Indians has reported lower levels of LDL-c associated with T allele (Saha et al., 1997). On the contrary, present study did not find any association LDL-c levels (among control subjects) even after using different genetic models. The factor that caused different results in these two studies could be the subject selection criteria. While both control cohorts of subjects were free of overt CAD, sampling in our study was performed in a fasting state as opposed the sampling in a non-fasting state in the previous study (Saha et al., 1997). Since Apo A-IV has a very short plasma residence time (Ghiselli, Krishnan, Beigel, & Gotto, 1986), it is more relevant in lipoprotein metabolism regulation in the postprandial state rather than in the fasting state where plasma Apo A-IV levels quickly fall to lower levels (Sherman & Weinberg,^1988^) which is turn possibly recedes serum LDL-c levels. This factor could have possibly caused the observed non-association of Apo AIV Thr347Ser polymorphism with LDL-c levels in the present study. Also, we in the present study have not studied the possible interaction of this SNP with another well-known SNP at codon 360 in the Apo AIV gene. It has been shown by Saha and colleagues in a two site haplotype analysis that there can be as much as twice the amount of LDL-c variation between the single site (codon 347) polymorphism and the double site (codon 347 and 360) polymorphism (i.e. 2.6 vs. 5.2% respectively) of the Apo AIV gene (Saha et al., 1997). Our study was however able to show lower levels of HDL-c among heterozygous mutants (AT) when compared to that in wild genotype (AA). This effect was however nullified as evident of the results of the comparison employing the dominant model, thus indicating that the positive association could be a statistical anomaly.

As per our knowledge, this is the first study among North Indians which has investigated the association of Thr347Ser polymorphism in the Apo AIV gene with CAD. Thus, from the present data, it would be safe to conclude that unlike the other ethnic populations of the world (especially Caucasians), North Indians tend to show no association of this SNP with CAD or fasting plasma cholesterol levels. However, this subject needs to be further investigated which warrants functional studies.

### 4.4. Genotypic/haplotypic combinations and CAD

We also assessed the role of all possible genotypic combinations arising due to Apolipoprotein AI (-75G > A), CIII-*SstI* and AIV Thr347Ser polymorphism with CAD. Interestingly using double genotype analysis we found that the frequency of combinations of Apo AI and AIV mutants were significantly higher in patients as opposed to controls, indicating that subjects carrying even one mutant alleles of both these polymorphisms carry at least 1.3-fold higher risk of developing CAD (*p* = 0.03, OR = 1.33, 95%CI = 1.06–1.65) (supplementary Table 2). Interestingly similar association was not seen in triple genotype analysis (supplementary Table 2). This association may well have been a “matter of chance”, as demonstrated by distribution of haplotype combinations which showed no significant difference among CAD patients and controls negating the role of haplotypic combinations of the selected polymorphisms in disease pathogenesis in the study population (Table 3).

Thus we prescribe a wholesome approach while calculating the composite risk of an individual. Genetic association studies may provide a better understanding of a “new factor”, adding to the knowledge of already known risk factors for a particular disease. Considering these conventional and newer risk factors together in risk assessment of an individual could ultimately help us in the future to correctly predict the onset of multifactorial diseases such as CAD.

## 5. Conclusion

To conclude, we have demonstrated association of these SNP’s with various cholesterol traits (among control population), but it seems that their extrapolated effect do not decisively/significantly contribute in the pathogenesis of CAD. Role of genotypic combinations of these SNP’s in the pathogenesis of CAD is still unclear and remains to be further evaluated. This suggests that there should be more data on these markers from different parts of India.

## Supplementary Material

Supplimentary_tables-2016.doc

## Supplementary material

The supplementary material for this paper is available online at http://dx.doi.10.1080/2331205X.2016.1266789.
