# Supplementary material for: Association of serum lipids and coronary artery disease with polymorphisms in the apolipoprotein AI-CIII-AIV gene cluster
Source: Cogent Med. 2016 Dec 17;3(1):1266789. doi: 10.1080/2331205X.2016.1266789 (PMC5314817; doi:10.1080/2331205X.2016.1266789)
Supplement: Supplimentary_tables-2016.doc [file oamd_a_1266789_sm8257.doc]

| **Supplementary table 1: PCR amplification and restriction digestion of SNP’s in ApoAI-CIII-AIV gene cluster** | | | | | | |
| --- | --- | --- | --- | --- | --- | --- |
| **Marker** | **Primers (5’-3’)** | **Annealing** | **Digestion** | **Allele** | **Product (bp)** | **Reference** |
| **ApoA-I (-75 G to A polymorphism)**  (rs1799837) | (F) AGGGACAGAGCTGATCCTTGAACTCTTAAG  (R) TTAGGGGACACCTAGCCCTCAGGAAGAGCA | 55°C for 120s and at 70°C for 120s | *Msp*I  37.0^0^C; 2h | A | 48, 207 | [9] |
|  |  |  |  | G | 110, 48 |  |
| **ApoC-III (SstI polymorphism)**  (3238C→G polymorphism) (rs5128) | (F) CATGGTTGCCTACAGGAGTTC  (R) TGACCTTCCGCACAAAGCTGT | 50.0 ^0^C; 60s | *Sst*I  37°C;  2h | S1(C) | 596 | [9] |
|  |  |  |  | S2(G) | 225, 371 |  |
| **ApoA-IV (T347S polymorphism)**  (rs675) | (F) GCCCTGGTGCAGCAGATGGAACAGCTCAGG  (R) CATCTGCACCTGCTCCTGCTGCTGCTCCAG | 65.0 ^0^C; 60s | *Hinf*I  37.0 ^0^C; 3h | Thr(A) | 150 | [25] |
|  |  |  |  | Ser(T) | 222 |  |

| **Supplementary table 2: Genotype combinations and their relation to CAD** | | | | | |
| --- | --- | --- | --- | --- | --- |
|  | **CAD patients(n=200)** | **Controls(n=200)** | **OR (95%CI)** | **χ^2^** | **p value** |
| **Double Genotype Analysis** |  |  |  |  |  |
| **Apo AI+CIII** |  |  |  |  |  |
| AI mutants^(AA or GA)^ + CIII mutants^(GG or CG)^ | 29(14.5%) | 21(10.5%) | 1.19(0.92-1.53) | 1.12 | 0.29 |
| AI mutants^(AA or GA)^ + CIII wild type^(CC)^ | 54(27.0%) | 61(30.5%) | 0.91(0.73-1.15) | 0.44 | 0.51 |
| AI wild type^(GG)^ + CIII mutants^(GG or CG)^ | 66(33.0%) | 67(33.5%) | 0.99(0.80-1.22) | 0.01 | 0.91 |
| AI wild type^(GG)^ + CIII wild type^(CC)^ | 51(25.5%) | 51(25.5%) | 1.00(0.80-1.25) | 0.00 | 1.00 |
| **Apo CIII+AIV** |  |  |  |  |  |
| CIII mutants^(GG or CG)^ + AIV mutants^(TT or AT)^ | 85(42.5%) | 75(37.5%) | 1.09(0.90-1.33) | 0.67 | 0.41 |
| CIII mutants^(CC or CG)^ + AIV wild type^(AA)^ | 10(5.0%) | 13(6.5%) | 0.86(0.53-1.39) | 0.18 | 0.67 |
| CIII wild type^(CC)^ + AIV mutants^(TT or AT)^ | 26(13.0%) | 19(9.5%) | 1.18(0.90-1.55) | 0.90 | 0.34 |
| CIII wild type^(CC)^ + AIV wild type^(AA)^ | 79(39.5%) | 93(46.5%) | 0.86(0.71-1.06) | 1.72 | 0.18 |
| **Apo AI+AIV** |  |  |  |  |  |
| AI mutants^(AA or GA)^ + AIV mutants^(TT or AT)^ | 41(20.5%) | 24(12.0%) | 1.33(1.06-1.65) | 4.70 | 0.03* |
| AI mutants^(AA or GA)^ + AIV wild type^(AA)^ | 42(21.0%) | 58(29.0%) | 0.80(0.62-1.03) | 3.00 | 0.08 |
| AI wild type^(GG)^ + AIV mutants^(TT or AT)^ | 70(35.0%) | 70(35.0%) | 1.00(0.81-1.23) | 0.00 | 1.00 |
| AI wild type^(GG)^ + AIV wild type^(AA)^ | 47(23.5%) | 48(24.0%) | 0.99(0.78-1.24) | 0.01 | 0.91 |
| **Triple Genotype analysis** |  |  |  |  |  |
| AI wild type^(GG)^ + CIII wild type^(CC)^ + AIV wild type^(AA)^ | 40(20.0%) | 38(19.0%) | 1.03(0.81-1.31) | 0.01 | 0.90 |
| AI mutants^(AA or GA)^ + CIII wild type^(CC)^ + AIV wild type^(AA)^ | 39(19.5%) | 55(27.5%) | 0.79(0.61-1.02) | 3.13 | 0.08 |
| AI mutants^(AA or GA)^ + CIII mutants^(CC or CG)^ + AIV wild type^(AA)^ | 3(1.5%) | 3(1.5%) | 1.00(0.45-2.24) | 0.00 | 1.00 |
| AI mutants^(AA or GA)^ + CIII mutants^(CC or CG)^ + AIV mutants^(TT or AT)^ | 26(13.0%) | 18(9.0%) | 1.21(0.92-1.58) | 1.25 | 0.26 |
| AI wild type^(GG)^ + CIII wild type^(CC)^ + AIV mutants^(TT or AT)^ | 11(5.5%) | 13(6.5%) | 0.91(0.58-1.42) | 0.04 | 0.83 |
| AI wild type^(GG)^ + CIII mutants^(CC or CG)^ + AIV mutants^(TT or AT)^ | 59(29.5%) | 57(28.5%) | 1.02(0.83-1.27) | 0.01 | 0.91 |
| AI wild type^(GG)^ + CIII mutants^(CC or CG)^ + AIV wild type^(AA)^ | 7(3.5%) | 10(5.0%) | 0.82(0.46-1.45) | 0.24 | 0.62 |
| AI mutants^(AA or GA)^ + CIII wild type^(CC)^ + AIV mutants^(TT or AT)^ | 15(7.5%) | 6(3.0%) | 1.46(1.09-1.95) | 3.22 | 0.07 |
| *p value of <0.05 was considered to be statistically significant | | | | | |
| Polymorphisms studied: Apo AI (-75 G/A polymorphism); Apo CIII (SSTL polymorphism); Apo AIV (T347S polymorphism) | | | | | |
